# *Desulfovibrio* Bacteremia in Patients with Abdominal Infections, Japan, 2020–2025

**DOI:** 10.3201/eid3202.251581

**Published:** 2026-02

**Authors:** Naoki Watanabe, Tomohisa Watari, Yoshihito Otsuka

**Affiliations:** Author affiliation: Kameda Medical Center, Kamogawa, Japan; 1 Current affiliation: Hirosaki University, Hirosaki, Japan.

**Keywords:** Desulfovibrio, bacteremia, bacteria, anaerobic, β-lactamase, blood culture, antimicrobial resistance, Japan, mass spectrometry, microbial sensitivity test

## Abstract

We reviewed 8 episodes of *Desulfovibrio* bacteremia in Japan (2020–2025) and confirmed 4 species by 16S rRNA gene and whole-genome sequencing. We detected β-lactamase genes in 2 *D. desulfuricans* (*bla*_DES-1_-like), 1 *D. falkowii* (*bla*_MUN-1_), and 2 *D. fairfieldensis* (*bla*_CfiA_-like). Mass spectometry failed to identify *D. falkowii* or *D. legallii*.

*Desulfovibrio* species bacteria are gram-negative, sulfate-reducing, obligately anaerobic curved or spiral rods that inhabit aquatic and soil environments, as well as the gastrointestinal tracts of humans and animals (*1*,*2*). Reported manifestations include bacteremia and intraabdominal infections, such as abscesses and cholecystitis (*2*). Several *Desulfovibrio* species have been implicated in human disease, including *D. desulfuricans*, *D. fairfieldensis*, *D. vulgaris*, and *D. piger* (*3*,*4*). *D. desulfuricans* is the most commonly reported species in *Desulfovibrio* bacteremia, which can result from translocation from the gastrointestinal tract (*2*). Species-level identification can be challenging in clinical laboratories. Routine matrix-assisted laser desorption/ionization time-of-flight (MALDI-TOF) mass spectrometry libraries might lack reference spectra for less commonly recognized species (*5*). As a result, routine identification may be uncertain, even at the genus level.

The optimal therapy for *Desulfovibrio* infections has not been determined as of January 2026. Reported isolates often show low MICs for metronidazole, whereas MICs for some β-lactams can be high; extended-spectrum β-lactamases, such as DES-1, have been described in *D. desulfuricans* (*6*,*7*). Species-resolved antimicrobial susceptibility data and resistance determinants remain limited beyond those for *D. desulfuricans* and *D. fairfieldensis*. We describe 8 episodes of *Desulfovibrio* bacteremia associated with abdominal infection in Japan and assess the limits of routine identification, confirming species by 16S rRNA gene and whole-genome sequencing. We also summarize patient characteristics, outcomes, antimicrobial MICs, and β-lactamase genes.

## The Study

We retrospectively reviewed clinical and laboratory data in cases of *Desulfovibrio* bacteremia at Kameda Medical Center, a tertiary-care hospital in Kamogawa, Japan, during January 2020–June 2025; we included episodes in which *Desulfovibrio* spp. were isolated from blood cultures. We considered all positive blood culture bottles collected during the same clinical episode as 1 case. We counted a new episode only when it was clearly associated with new symptoms or signs, a new anatomic focus, or resolution of a previous episode. We assessed outcomes during hospitalization, which we defined as the period from initiation of antimicrobial therapy through hospital discharge. The ethics committee of Kameda Medical Center approved the study (approval no. 25-061) and waived the requirement for informed consent because of the retrospective study design and use of deidentified data.

We processed blood cultures with the BACTEC FX system (Becton, Dickinson and Company, https://www.bd.com) and incubated them at 35°C for <7 days in accordance with our routine protocol. We defined the time to positivity as the interval from the start of incubation to the first instrument-flagged positive bottle. We performed routine identification by MALDI-TOF mass spectrometry and a desulfoviridin assay. We performed species identification with a MALDI Biotyper using the MBT Compass Library version 13 (Bruker Daltonics GmbH, https://www.bruker.com). We considered scores >2.0 as species-level identifications and scores <2.0 as uncertain. We confirmed species by 16S rRNA gene and whole-genome sequencing (Appendix Tables 1, 2). For whole-genome sequencing, we generated paired-end reads on an Illumina MiSeq instrument (Illumina, https://www.illumina.com), assembled reads de novo, evaluated assembly quality, and assigned species by comparing average nucleotide identity with type strains and reference genomes. We identified antimicrobial drug resistance determinants from draft assemblies and determined antimicrobial drug susceptibility by microdilution in Brucella broth on dry plates (Eiken Chemical, https://www.eiken.co.jp) incubated anaerobically at 35°C–37°C for 48–96 hours. (Appendix).

We identified 8 episodes of *Desulfovibrio* bacteremia among 4,431 patients with positive blood cultures (0.2% [95% CI 0.1%–0.4%]). All patients were >65 years of age (median 81 years; interquartile range 77–86 years); the presumed source in 7 episodes was abdominal infection (Table 1). The primary initial symptoms were fever (4/8) and abdominal symptoms (5/8) (Appendix Table 3). Median time to positivity was 4.1 days (range 2.9–5.5 days), and 3 episodes became positive on incubation day 6 (Table 1). We assessed outcomes at discharge; 1 patient died. Case 7 was considered of uncertain clinical significance because symptoms had resolved without antimicrobial therapy by the time of culture notification and the patient declined further evaluation; he was later confirmed to be alive when he returned for care for an unrelated illness ≈1 year later.

**Table 1:** Patient characteristics and clinical course of *Desulfovibrio* bacteremia in patients with abdominal infections, Japan, 2020–2025

Case no.	Age, y/sex	Clinical diagnosis	Underlying conditions	Time to positivity, d	Source control	Antimicrobial therapy (duration, d)*	Outcome at discharge
1†	80s/M	Ischemic colitis, bowel obstruction	Atrial fibrillation; hypertension	4	Yes (surgery)	Ampicillin/sulbactam (9), then amoxicillin/clavulanate (7)	Recovered
2	70s/M	Ischemic colitis, septic shock	Intravascular large B cell lymphoma; chronic hepatitis B; hypertension	5	No	Piperacillin/tazobactam, vancomycin, and micafungin (3), then piperacillin/tazobactam (11)	Recovered
3	80s/F	Adhesive small-bowel obstruction; hemorrhagic cystitis	Ureteral cancer; severe aortic stenosis; paroxysmal atrial fibrillation	6	No	Cefotiam (5), then ampicillin/sulbactam (14)	Recovered
4	60s/F	Perianal abscess	Rectal cancer; prior venous thromboembolism; recent colostomy	6	Yes (surgery)	Piperacillin/tazobactam (30), then piperacillin and metronidazole (3)	Recovered
5	80s/M	Psoas abscess; catheter-associated urinary tract infection	Aortic stenosis/aortic regurgitation; atrial fibrillation; heart failure	3	No	Cefepime and vancomycin (7)	Died
6	90s/M	Colonic diverticulitis; acute enteritis	None	3	No	Piperacillin/tazobactam (duration not recorded)	Transferred
7	70s/M	Febrile illness, unknown origin	Hypertension	6	No	None	Not applicable‡
8	70s/M	Colonic diverticulitis	Hypertension	4	No	Amoxicillin/clavulanate (10)	Recovered

*Antimicrobial agents are listed as the drugs used for the longest duration. When the regimen changed, the initial regimen is listed first, followed by subsequent therapy. †Polymicrobial episode with co-pathogen *Parabacteroides goldsteinii*. ‡Indicates an episode of uncertain clinical significance that was not managed as clinically significant bacteremia. The patient declined further evaluation and was confirmed to be alive at ≈1-y follow-up.

**Figure 1:**
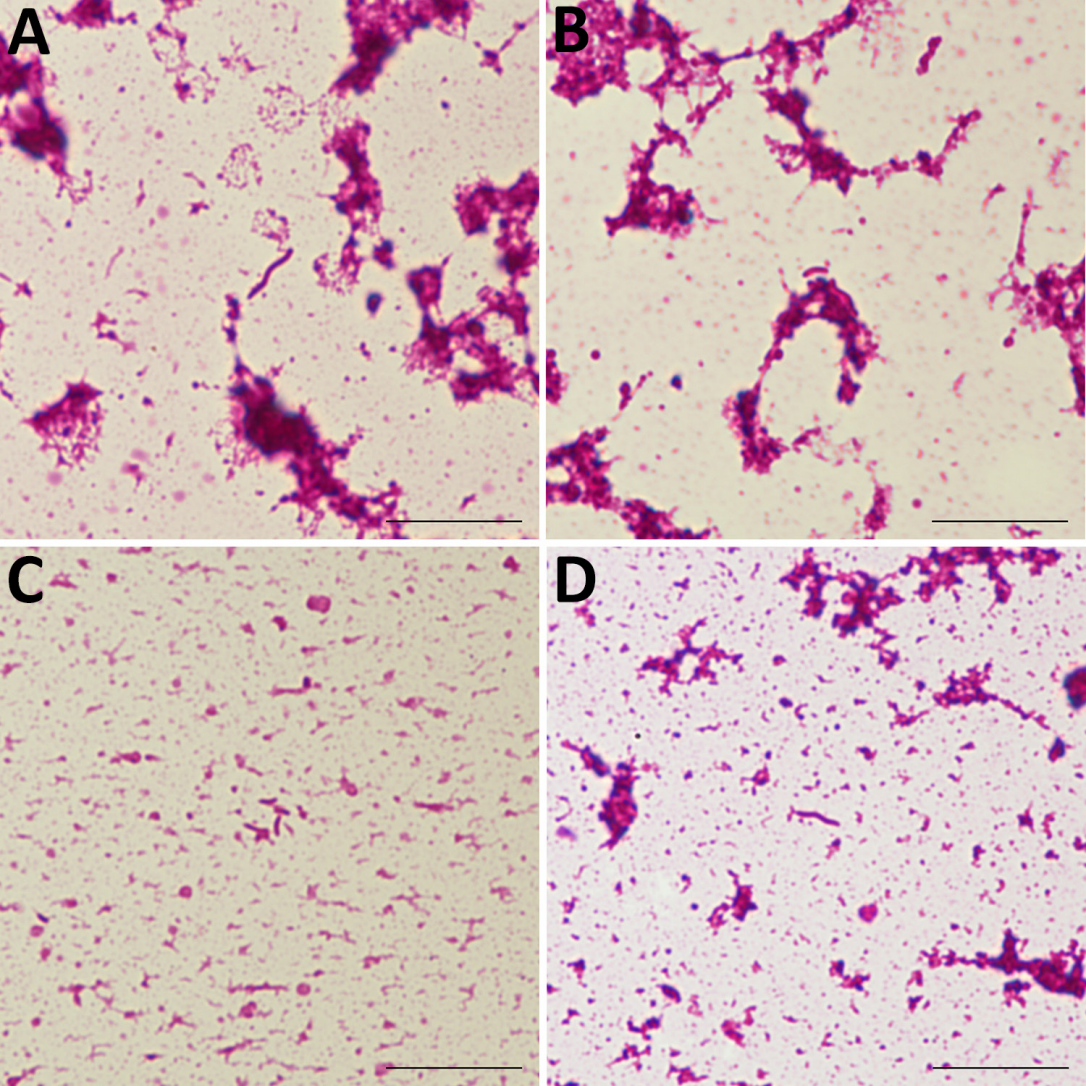
Gram-stained smear from a positive anaerobic blood-culture bottle in a study of *Desulfovibrio* bacteremia at a tertiary-care hospital in Japan, 2020–2025. A) *D. desulfuricans* spiral form; B) *D. desulfuricans* curved form; C) *D. falkowii* curved form; D) *D. legallii* spiral form. Curved or spiral gram-negative rods are visible. Images were acquired using a 100× oil-immersion objective. Scale bars indicate 10 μm.

**Figure 2:**
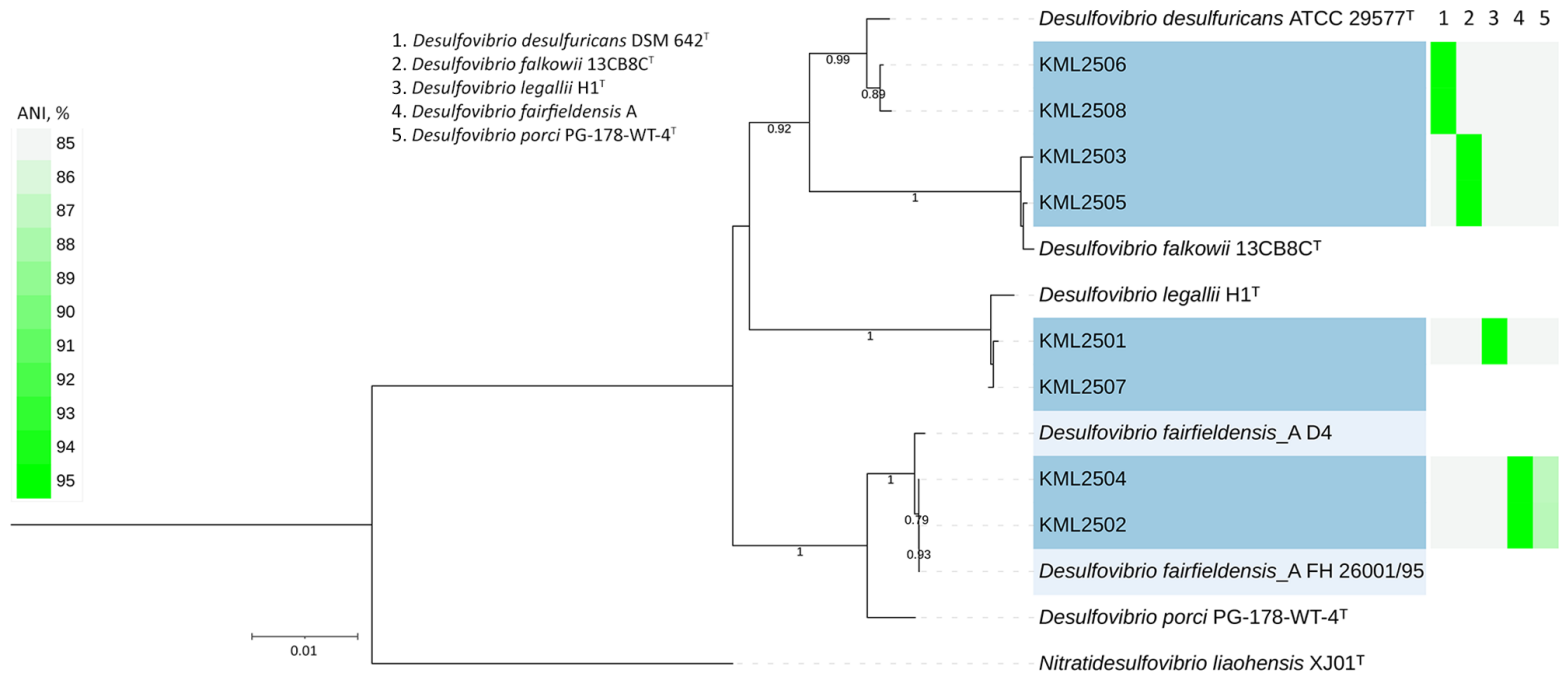
Phylogenetic tree of 8 *Desulfovibrio* isolates from study of *Desulfovibrio* bacteremia at a tertiary-care hospital in Japan, 2020–2025. Tree is based on 16S rRNA gene sequences and ANI heatmap and dendrogram comparing study isolates (numbers at left, defined in the key) with type or reference genomes. Cells are colored by ANI (%) on a fluorescent-green gradient; higher identity appears brighter. Blue shading indicates study isolates. Light blue shading indicates previously reported *D. fairfieldensis* isolates (FH 26001/95 and D4). Superscript T indicates type strains. Scale bar indicates number of substitutions per site. ANI, average nucleotide identity.

We identified 2 isolates each of *D. desulfuricans*, *D. fairfieldensis*, *D. falkowii*, and *D. legallii* (Table 2). Gram stains from anaerobic blood culture bottles showed curved gram-negative rods (Figure 1). The desulfoviridin assay was positive for all isolates. Seven isolates passed genome quality thresholds and were assigned to species by average nucleotide identity and digital DNA–DNA hybridization (Figure 2; Appendix Tables 4–8). The remaining isolate was identified as *D. legallii* by 16S rRNA gene sequencing because its genome assembly did not meet completeness criteria. Previous reports emphasized *D. desulfuricans* and *D. fairfieldensis* as predominant causes of bacteremia (*2*), whereas our series also included *D. falkowii* and *D.* *legallii*. Bacteremia caused by *D. falkowii* or *D. legallii* has been reported infrequently (*5*,*8*). MALDI-TOF mass spectrometry did not identify *D. falkowii* or *D. legallii* (Table 2); that finding was consistent with a previous report of *D. legallii* bacteremia in which MALDI-TOF mass spectrometry failed to identify the species (*5*). Sequence data are available in DDBJ/GenBank under BioProject PRJDB35884 (Appendix Table 9).

**Table 2:** Characteristics of *Desulfovibrio* isolates in patients with abdominal infections, Japan, 2020–2025*

Case no.	Confirmed species (method)	MALDI-TOF MS primary result (score)	MIC, μg/mL					β-lactamase genes detected
			SAM	TZP	FOX	CRO	MTZ	
1	*D. legallii* (WGS/ANI)	Uncertain (<2.0)	≤0.5	>64	>32	4	<0.5	None
2	*D. fairfieldensis* (WGS/ANI)	*D. fairfieldensis* (2.4)	16	>64	>32	>32	<0.5	*bla*_CfiA_-like
3	*D. falkowii* (WGS/ANI)	Uncertain (<2.0)	<0.5	64	>32	8	<0.5	None
4	*D. fairfieldensis* (WGS/ANI)	*D. fairfieldensis* (2.4)	8	>64	>32	>32	<0.5	*bla*_CfiA_-like
5	*D. falkowii* (WGS/ANI)	Uncertain (<2.0)	4	64	>32	>32	<0.5	*bla*_MUN-1_
6	*D. desulfuricans* (WGS/ANI)	*D. desulfuricans* (2.1)	1	32	>32	32	<0.5	*bla*_DES-1_-like
7†	*D. legallii* (16S)	Uncertain (<2.0)	1	64	>32	8	<0.5	Not determined
8	*D. desulfuricans* (WGS/ANI)	*D. desulfuricans* (2.1)	2	64	>32	>32	<0.5	*bla*_DES-1_-like

*MICs were determined by broth microdilution in *Brucella* broth under anaerobic conditions. Resistance genes were identified in draft genomes by using AMRFinderPlus (https://github.com/ncbi/amr). ANI, average nucleotide identity; CRO, ceftriaxone; FOX, cefoxitin; MALDI-TOF MS, matrix-assisted laser desorption/ionization time-of-flight mass spectrometry; MTZ, metronidazole; SAM, ampicillin/sulbactam; TZP, piperacillin/tazobactam; WGS, whole-genome sequencing. †For case 7, β-lactamase gene detection was not determined because the genome assembly did not meet completeness criteria.

Antimicrobial susceptibility testing by broth microdilution showed low MICs for ampicillin/sulbactam and metronidazole (Table 2; Appendix Table 10). Piperacillin/tazobactam MICs were 32 to >64 μg/mL for all isolates (Table 2), consistent with previous observations (*6*,*9*). We did not perform Etest susceptibility testing (bioMérieux, https://www.biomerieux.com), so could not assess agreement with broth microdilution MICs. Five isolates had ceftriaxone MICs ≥32 μg/mL and carried β-lactamase genes, including *bla*_DES-1_-like, *bla*_MUN-1_, or *bla*_CfiA_-like (Table 2), suggesting that β-lactamase activity contributes to elevated ceftriaxone MICs in some isolates. DES-1 has been described in *D. desulfuricans* (*7*). MUN-1 is an Ambler class A extended-spectrum β-lactamase (*10*). CfiA-family class B metallo-β-lactamases have been described in the *Bacteroides fragilis* group (*11*). *D. falkowii* isolate KML2505 carried *bla*_MUN-1_ with 100% identity and 100% coverage to reference isolate WP_206340447.1. *D. desulfuricans* isolates KML2506 and KML2508 each carried DES-family class A β-lactamases (*bla*_DES-1_-like) with 81%–82% identity and 100% coverage to the closest reference WP_063860095.1 isolate. In addition, *D. fairfieldensis* isolates KML2502 and KML2504 harbored subclass B1 metallo-β-lactamase homologs (*bla*_CfiA_-like) with 47% identity and 94% coverage to the closest reference, WP_005808062.1.

## Conclusions

In this case series, *Desulfovibrio* bacteremia was associated with multiple species, including *D. desulfuricans*, *D. fairfieldensis*, *D. falkowii*, and *D. legallii*, suggesting broader species diversity than previously appreciated. Antimicrobial drug susceptibility testing showed low MICs for metronidazole and ampicillin/sulbactam, whereas MICs for piperacillin/tazobactam were high in all isolates. Routine MALDI-TOF mass spectrometry did not identify *D. falkowii* or *D. legallii* bacteria. Curved gram-negative rods in anaerobic blood culture bottles and a positive desulfoviridin assay may prompt suspicion for *Desulfovibrio* infection, which can guide empiric therapy while confirmatory identification is pending.

Appendix Additional information about *Desulfovibrio* bacteremia in patients with abdominal infections, Japan, 2020–2025.
