# Supplementary material for: Desulfovibrio Bacteremia in Patients with Abdominal Infections, Japan, 2020–2025
Source: Emerg Infect Dis. 2026 Feb;32(2):233–7. doi: 10.3201/eid3202.251581 (PMC12928213; doi:10.3201/eid3202.251581)
Supplement: Appendix — Additional information about Desulfovibrio bacteremia in patients with abdominal infections, Japan, 2020–2025. [file 25-1581-Techapp-s1.pdf]

Article DOI: https://doi.org/10.3201/eid3202.251581

EID cannot ensure accessibility for supplementary materials supplied by authors. Readers who have difficulty accessing supplementary content should contact the authors for assistance.

Desulfovibrio Bacteremia in Patients with Abdominal Infections, Japan, 2020–2025

Appendix

Methods

Blood Culture Processing and Time to Positivity

Blood cultures were processed with the BD BACTEC FX system (BD, https://www.bd.com). We used BACTEC Plus Aerobic/F and Lytic/10 Anaerobic/F bottles for adults. Bottles were incubated at 35°C for up to 7 days under our routine protocol. We defined time to positivity as the interval from the start of incubation after bottle loading to the first instrument-flagged positive bottle. For each episode, we used the earliest positive time among positive bottles. Time-to-positivity values were obtained from the instrument log.

Isolate Storage and Subculture Conditions

Desulfovibrio isolates were stored at −80°C. Before testing, isolates were subcultured on Brucella HK agar (Kyokuto Pharmaceutical Industrial, https://www.kyokutoseiyaku.co.jp) and incubated anaerobically at 35–37°C for 4–6 days using the AnaeroPack system (Mitsubishi Gas Chemical Company, https://www.mgc.co.jp).

Antimicrobial Susceptibility Testing

Antimicrobial susceptibility was determined by broth microdilution in Brucella broth using dry plates (Eiken Chemical, https://www.eiken.co.jp). Inoculates were prepared according to the manufacturer's instructions and dispensed at a final concentration of 1 × 10^5 CFU per well. Plates were incubated anaerobically at 35–37°C for 48 h. If growth was insufficient in control well, incubation was extended up to 96 h. After confirming adequate growth in the controls, we read MICs as the lowest concentrations without visible growth. We tested penicillin, ampicillin/sulbactam, amoxicillin/clavulanate, piperacillin/tazobactam, ceftriaxone, cefoxitin, imipenem, clindamycin, moxifloxacin, and metronidazole. Because interpretive breakpoints have

not been established for *Desulfovibrio* by broth microdilution, we report MICs in μg/mL without categorizing isolates as susceptible or resistant.

Desulfoviridin Assay

Apply 2 N NaOH to the swabbed colony or broth sediment and examine immediately under 365-nm UV in a dark box; red fluorescence indicates a positive result.

16S rRNA Gene Sequencing and Analysis

We extracted genomic DNA using a MagLEAD instrument and the manufacturer's kit (Precision System Science, https://www.pss.co.jp). We amplified the 16S rRNA gene by PCR using primers listed in Appendix Table 1. For PCR amplification, we prepared a 50-μL reaction mixture consisting of 2 μL of template DNA, 25 μL of Premix Taq Hot Start Version (Takara Bio, https://www.takara-bio.com), 2 μL each of forward and reverse primers, and 19 μL of distilled water. Primers were synthesized by FASMAC (https://fasmac.co.jp). PCR was performed using a GeneAtlas Type G thermal cycler (Astec, https://www.astec-corp.co.jp). Sanger sequencing was outsourced to FASMAC.

We trimmed the sequences and queried them using BLASTn (https://blast.ncbi.nlm.nih.gov), restricting the search set to type material. For 16S-based species assignment, percent identity ≥99.0% was considered supportive evidence for species-level assignment.

For phylogenetic analysis, we performed multiple sequence alignment using MUSCLE in MEGA version 12 (https://www.megasoftware.net). We constructed a neighbor-joining tree using the Kimura 2-parameter model, pairwise deletion of gaps, and 100 bootstrap replicates. Using a prespecified ≥1,300-bp length threshold, we reanalyzed previously reported 16S sequences labeled as *Desulfovibrio fairfieldensis* and included eligible sequences alongside study isolates and type strain sequences. Trees were visualized and annotated using Interactive Tree of Life (iTOL) version 7 (https://itol.embl.de).

Whole-Genome Sequencing and Analysis

We prepared sequencing libraries using Illumina DNA Prep (M) Tagmentation (Illumina, https://www.illumina.com) and generated paired-end reads on an Illumina MiSeq instrument. We assembled reads de novo using the Bacterial and Viral Bioinformatics Resource Center (BV-

BRC) platform (https://www.bv-brc.org). We evaluated genome completeness and contamination using CheckM (*1*).

We annotated draft assemblies and performed species assignments using average nucleotide identity (ANI) computed with fastANI by comparison with type strain and reference genomes (*2*). For isolates assigned to *D. fairfieldensis*, we also computed digital DNA–DNA hybridization (dDDH) using the Genome-to-Genome Distance Calculator (https://ggdc.dsmz.de) (*3*). We identified antimicrobial resistance determinants from draft assemblies using AMRFinderPlus (*4*).

References

1. Parks DH, Imelfort M, Skennerton CT, Hugenholtz P, Tyson GW. CheckM: assessing the quality of microbial genomes recovered from isolates, single cells, and metagenomes. Genome Res. 2015;25:1043–55. PubMed https://doi.org/10.1101/gr.186072.114

2. Jain C, Rodriguez-R LM, Phillippy AM, Konstantinidis KT, Aluru S. High throughput ANI analysis of 90K prokaryotic genomes reveals clear species boundaries. Nat Commun. 2018;9:5114. PubMed https://doi.org/10.1038/s41467-018-07641-9

3. Meier-Kolthoff JP, Auch AF, Klenk HP, Göker M. Genome sequence-based species delimitation with confidence intervals and improved distance functions. BMC Bioinformatics. 2013;14:60. PubMed https://doi.org/10.1186/1471-2105-14-60

4. Feldgarden M, Brover V, Haft DH, Prasad AB, Slotta DJ, Tolstoy I, et al. Validating the AMRFinder tool and resistance gene database by using antimicrobial resistance genotype–phenotype correlations in a collection of isolates. Antimicrob Agents Chemother. 2019;63:e00483-19. PubMed https://doi.org/10.1128/AAC.00483-19

Appendix Table 1. Primers for 16S rRNA gene amplification and sequencing

Primer name	Sequence (5′→3′)	Orientation
8F	AGAGTTTGATCCTGGCTCAG	Forward
337F	GACTCCTACGGGAGGCWGCAG	Forward
518R	GTATTACCGCGGCTGCTGG	Reverse
928F	TAAAACTYAAAKGAATTGACGGG	Forward
1492R	CGGTTACCTTGTTACGACTT	Reverse

Appendix Table 2. Tools used for whole-genome analysis*

Tool	Purpose	References	Notes
CheckM v1.2.3	Genome quality checks	1	Genomes with completeness <90% or contamination ≥5% were excluded from downstream analyses*.
fastANI v1.34	Average nucleotide identity (ANI)	2	ANI ≥95% was interpreted as the same species
Genome–Genome Distance Calculator 3.0	Digital DNA-DNA hybridization (dDDH)	3	We used the recommended formula. Species-level relatedness was assigned at dDDH ≥70%.
AMRFinderPlus v4.0.23	Antimicrobial resistance determinants	4	Standard thresholds were applied to classes other than β-lactamases, with the homology threshold changed to ≥30% for β-lactamases to capture distantly related homologs.

*Because CheckM reported 7.7% contamination even for the *D. desulfuricans* DSM 642^{T} genome (GCA_000420465.1), we accepted contamination ≤10% for species assignment and ANI analyses within the *D. desulfuricans* lineage.

Appendix Table 3. Detailed clinical variables and comorbidity data*

Characteristics	No. (%), n = 8
Comorbid conditions	
Myocardial infarction	0
Congestive heart failure	3 (37.5)
Peripheral vascular disease	1 (12.5)
Cerebrovascular disease	1 (12.5)
Dementia	0
Chronic pulmonary disease	1 (12.5)
Connective tissue disease	0
Peptic ulcer disease	2 (25.0)
Mild liver disease	1 (12.5)
Moderate or severe liver disease	0
Uncomplicated diabetes mellitus	0
Diabetes mellitus with end-organ damage	0
Hemiplegia or hemiparesis	0
Renal disease	0
Non-metastatic solid tumor	2 (25.0)
Metastatic solid tumor	0
Leukemia	0
Malignant lymphoma	1 (12.5)
Immunocompromised conditions	
HIV	0
Chemotherapy	1 (12.5)
Long-term corticosteroid use	1 (12.5)
Immunosuppressive therapy	0
Organ or stem cell transplantation	0
Febrile neutropenia within 7 d	0
Indwelling devices within 30 d	4 (50.0)
Symptoms	
Fever	4 (50.0)
Abdominal pain	4 (50.0)
Abdominal distension	1 (12.5)
Obstipation	2 (25.0)

*Data are no. (%) unless otherwise indicated.

Appendix Table 4. Genome statistics of *Desulfovibrio* isolates*

Isolate ID	Genome size, Mb	Contigs, n	Longest contig, bp	N50, bp	GC content, %	CDS, n	Coding ratio, %	rRNA, n	tRNA, n	CRISPR, n	Completeness, %	Contamination, %
KML2501	2.76	30	574,094	265,214	62.4	2,331	85.7	3	47	2	99.11	0.01
KML2502	3.43	45	447,596	215,787	61.4	2,876	84.8	3	51	1	99.11	0.00
KML2503	3.24	51	476,950	138,177	57.7	2,806	84.1	3	48	1	98.96	0.00
KML2504	3.75	77	353,551	164,050	60.8	3,275	84.4	3	48	0	98.96	0.22
KML2505	3.00	31	518,440	214,732	58.0	2,532	84.1	3	48	1	98.96	0.00
KML2506	3.54	15	868,136	358,750	57.2	3,060	86.1	3	54	1	93.68	9.60
KML2507	NA	NA	NA	NA	NA	NA	NA	NA	NA	NA	20.61	0.00
KML2508	3.54	94	300,804	148,734	57.4	3,101	85.1	3	53	1	92.71	10.43

*Genome size is the total length of assembled contigs. KML2507 did not meet inclusion criteria and is listed as NA except for the reported completeness and contamination. CDS, coding sequences; NA, not available.

Appendix Table 5. 16S rRNA BLAST results (restricted to type material)*

Isolate ID	Closest match (accession no.)	Percent identity	Identities, n/N	Query coverage, %	E-value
KML2501	*Desulfovibrio legallii* H1^T (NR_108301.1)	99.5	1,435/1,443	99	0.0
KML2502	*Desulfovibrio porci* PG-178-WT-4^T (MN537481.1)	98.99	1,466/1,481	100	0.0
KML2503	*Desulfovibrio falkowii* 13CB8C^T (NR_199409.1)	99.8	1,478/1,481	100	0.0
KML2504	*Desulfovibrio porci* PG-178-WT-4^T (MN537481.1)	98.99	1,468/1,483	100	0.0
KML2505	*Desulfovibrio falkowii* 13CB8C^T (NR_199409.1)	99.9	1,481/1,483	100	0.0
KML2506	*Desulfovibrio desulfuricans* Essex 6 (NR_104990.1)	99.7	1,473/1,478	100	0.0
KML2507	*Desulfovibrio legallii* H1^T (NR_108301.1)	99.5	1,435/1,443	97	0.0
KML2508	*Desulfovibrio desulfuricans* Essex 6 (NR_104990.1)	99.5	1,471/1,478	100	0.0
FH 26001/95	*Desulfovibrio porci* PG-178-WT-4^T (MN537481.1)	99.0	1,466/1,481	98	0.0
D4	*Desulfovibrio porci* PG-178-WT-4^T (MN537481.1)	99.0	1,475/1,490	97	0.0

*Superscript T indicates type strains.

Appendix Table 6. Sequence data used for phylogenomic analyses

Type strain number	Strain	Data type	Accession no.
D. desulfuricans	ATCC 29577^T	16S	AF192153.1
D. falkowii	13CB8C^T	16S	LC776912.1
D. legallii	H1^T	16S	FJ225426.1
D. porci	PG-178-WT-4^T	16S	MN537481.1
D. fairfieldensis	FH 26001/95	16S	U42221.1
D. fairfieldensis	D4	16S	AF192155.1
NitratiDesulfovibrio liaohensis	XJ01^T	16S	MK260014.1
D. desulfuricans	DSM 642^T	WGS	GCA_000420465.1
D. falkowii	13CB8C^T	WGS	GCA_045865545.1
D. legallii	H1^T	WGS	GCA_004309735.1
D. porci	PG-178-WT-4^T	WGS	GCA_009696265.1
D. fairfieldensis	CCUG 45958^T	WGS	GCA_001553605.1

*Superscript T indicates type strains.

Appendix Table 7. Average nucleotide identity (%) to reference genomes

Isolate	*D. desulfuricans* DSM 642^T	*D. falkowii* 13CB8C^T	*D. legallii* H1^T	*D. fairfieldensis* CCUG 45958	*D. porci* PG-178-WT-4^T
KML2501	79.2	79.3	**99.4**	80.1	80.2
KML2502	79.3	79.7	80.2	**98.8**	87.4
KML2503	79.2	**99.2**	78.9	79.7	78.8
KML2504	79.1	79.8	80.2	**98.7**	87.3
KML2505	79.2	**99.4**	78.7	78.7	78.8
KML2506	**95.5**	79.1	79.4	79.2	79.1
KML2508	**95.6**	79.2	79.4	79.3	79.2

*Superscript T indicates type strains. Bold text indicates ANI $\geq$95%. ANI, average nucleotide identity.

Appendix Table 8. Digital DNA–DNA hybridization results*

Isolate ID	Reference species	Reference accession no.	dDDH, %	95% CI	Prob of dDDH $\geq$70%, (%)	G+C difference, %
KML2502	*D. fairfieldensis*	GCF_001553605.1	91.1	88.9 - 92.9	96.15	0.52
KML2502	*D. porci*	GCA_009696265.1	31.7	29.3 - 34.2	0.21	0.14
KML2504	*D. fairfieldensis*	GCF_001553605.1	90.0	87.7 - 91.9	95.81	0.08
KML2504	*D. porci*	GCA_009696265.1	31.6	29.2 - 34.2	0.2	0.74

*Digital DNA–DNA hybridization (dDDH) was computed with GGDC 3.0 (Formula 2; BLAST+). Confidence intervals and probabilities are those provided by GGDC. *D. fairfieldensis* denotes a GTDB placeholder species lacking valid publication and a type strain; *D. porci* is the validly published type comparison.

Appendix Table 9. Accessions and metadata for study isolates

Isolate ID	Whole-genome assembly accession no.	16S rRNA accession no.
KML2501	BAAIIN010000001-BAAIIN010000030	LC894614
KML2502	BAAIIO010000001-BAAIIO010000045	LC894615
KML2503	BAAIIP010000001-BAAIIP010000051	LC894616
KML2504	BAAIIQ010000001-BAAIIQ010000077	LC894617
KML2505	BAAIIR010000001-BAAIIR010000031	LC894618
KML2506	BAAIIS010000001-BAAIIS010000015	LC894619
KML2507	Not applicable	LC894620
KML2508	BAAIIT010000001-BAAIIT010000094	LC894621

Appendix Table 10. Antimicrobial susceptibility results of *Desulfovibrio* isolates*

Isolate ID	Species	PEN	SAM	AMC	TZP	CRO	FOX	IPM	CLI	MXF	MTZ
KML2506	*D. desulfuricans*	>1	1	1	32	32	>32	2	0.5	>4	≤0.5
KML2508	*D. desulfuricans*	>1	2	0.5	64	>32	>32	4	>4	4	≤0.5
KML2503	*D. falkowii*	1	≤0.5	0.25	64	8	>32	≤0.25	0.5	>4	≤0.5
KML2505	*D. falkowii*	>1	4	4	64	>32	>32	4	2	>4	≤0.5
KML2502	*D. fairfieldensis*	>1	16	>8	>64	>32	>32	>8	0.5	1	≤0.5
KML2504	*D. fairfieldensis*	>1	8	8	>64	>32	>32	4	0.5	0.5	≤0.5
KML2501	*D. legallii*	>1	≤0.5	0.5	>64	4	>32	2	0.5	>4	≤0.5
KML2507	*D. legallii*	>1	1	0.5	64	8	>32	1	0.25	>4	≤0.5

* Data are MICs (MICs, µg/mL). AMC, amoxicillin/clavulanate; CLI, clindamycin; CRO, ceftriaxone; FOX, cefoxitin; IPM, imipenem; MTZ, metronidazole; MXF, moxifloxacin; PEN, penicillin; SAM, ampicillin/sulbactam; TZP, piperacillin/tazobactam.
